# Axonal Selectivity of Myelination by Single Oligodendrocytes Established During Development in Mouse Cerebellar White Matter

**DOI:** 10.1002/glia.24660

**Published:** 2024-12-17

**Authors:** Batpurev Battulga, Yasuyuki Osanai, Reiji Yamazaki, Yoshiaki Shinohara, Nobuhiko Ohno

**Affiliations:** ^1^ Division of Histology and Cell Biology, Department of Anatomy, School of Medicine Jichi Medical University Shimotsuke Japan; ^2^ Department of Anatomy and Systems Biology, Faculty of Medicine University of Yamanashi Chuo Japan; ^3^ Division of Ultrastructural Research National Institute for Physiological Sciences Okazaki Japan

**Keywords:** cerebellum, myelin, neurodevelopment, oligodendrocyte, Purkinje cell

## Abstract

Myelin formation by oligodendrocytes regulates the conduction velocity and functional integrity of neuronal axons. While individual oligodendrocytes form myelin sheaths around multiple axons and control the functions of neural circuits where the axons are involved, it remains unclear if oligodendrocytes selectively form myelin sheaths around specific subtypes of axons. Using the combination of rabies virus‐mediated single oligodendrocyte labeling and immunostaining with tissue clearing, we revealed that approximately half of the oligodendrocytes preferentially myelinate axons originating from Purkinje cells in the white matter of adult mouse cerebella. The preference for Purkinje cell axons was more pronounced during development when the process of myelination within cerebellar white matter was initiated; over 90% of oligodendrocytes preferentially myelinated Purkinje cell axons. Preferential myelination of Purkinje cell axons was further confirmed by immuno‐electron microscopy and transgenic mice that label early‐born oligodendrocytes. Transgenic mice that label oligodendrocytes differentiated at the early development showed that early‐born oligodendrocytes preferentially myelinate Purkinje cell axons in the matured cerebellar white matter. In contrast, transgenic mice that label oligodendrocytes differentiated after the peak of cerebellar myelination showed that the later‐differentiated oligodendrocytes dominantly myelinated non‐Purkinje cell axons. These results demonstrate that a significant proportion of oligodendrocytes preferentially myelinate functionally distinct axons in the cerebellar white matter, and the axonal preference of myelination by individual oligodendrocytes is established depending on the timing of their differentiation during development. Our data provide the evidence that there is a critical time window of myelination that a specific subtype of axons are dominantly myelinated by the oligodendrocytes.

## Main Points

- Nearly half of OLs preferentially myelinate specific types of axons in the cerebellar white matter of adult mice.
- Early and later differentiated oligodendrocytes dominantly myelinate Purkinje cell and non‐Purkinje cell axons, respectively.

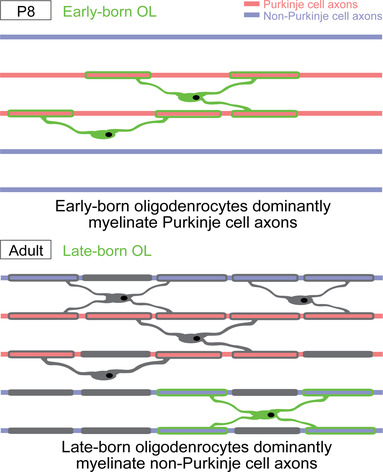

## 1 Introduction

Oligodendrocytes form myelin sheaths, which increase axonal conduction velocities and are critical for brain functions. It has been established that oligodendrocytes play key roles in maintaining the functional integrity of myelinated axons (Nave 2010). Given that each oligodendrocyte extends multiple processes and forms myelin sheaths around multiple axons, oligodendrocytes would regulate the functions of the neural circuits, in which the myelinated axons are involved. Previous studies examined the subtypes of axons myelinated by individual oligodendrocytes. In the cerebral cortex, examination using volume electron microscopy suggested that some oligodendrocytes disproportionately myelinate inhibitory interneuron axons, whereas others primarily target excitatory axons or show no bias (Zonouzi et al. 2019). In addition, in the corpus callosum, analyses performed using volume electron microscopy indicated that individual oligodendrocytes formed compact myelin with similar thicknesses preferentially around distant axons with a limited range of diameters (Tanaka et al. 2021). These previous studies support the concept that individual oligodendrocytes preferentially myelinate specific types of axons, which can be discriminated by structural properties and/or axonal functions (Yang et al. 2020). However, it remains unclear how such preference is generated in the white matter of the brain.

The cerebellum orchestrates complex motor functions, cognitive processes, and balance coordination (Ito 2008). Cerebellar white matter involves three types of axons (Voogd and Glickstein 1998). One of them is inhibitory Purkinje cell axons, which correspond to the output of the cerebellar cortex. Others include excitatory climbing fibers and mossy fibers, which are the two major input pathways toward the cerebellar cortex. The input and output fibers of the cerebellar cortex pass through the cerebellar white matter and are myelinated by adulthood (Nguyen et al. 2018). Motor functions associated with the cerebellum can be severely affected in demyelination and dysmyelination involving the cerebellar white matter (Uchida et al. 2012; Wilkins 2017). It is known that damage to the cerebellar white matter during the neonatal period can consequently result in motor and intellectual disabilities later in life. However, little is known about the myelin formation by each oligodendrocyte toward a particular subtype of axons in each developmental stage.

In this study, we aimed to address the preference for specific types of axons by individual oligodendrocytes in the cerebellar white matter. To achieve this goal, we used immunostaining for axonal markers and tissue clearing combined with the attenuated rabies virus that sparsely labels oligodendrocytes and transgenic animals that label early or later‐born oligodendrocytes, together with serial immune‐electron microscopic analysis. Our results demonstrated that a substantial proportion of oligodendrocytes preferentially myelinated Purkinje cell axons, and this preference was derived from selective myelination by oligodendrocytes that differentiated during the initial stage of myelination.

## 2 Materials and Methods

### 2.1 Animals and Tamoxifen Injection

C57BL/6 mice were purchased from Japan SLC (Shizuoka, Japan). *PLP‐CreERT* mice (Doerflinger, Macklin, and Popko 2003) and *Tau‐mGFP* mice (Hippenmeyer et al. 2005) were obtained from the Jackson Laboratory (JAX stock # 005975 and # 021162, respectively). *PDGFRa‐CreERT2* mice were gifted by Dr. William D Richardson (University College London) (Rivers et al. 2008). *PLP‐CreERT* and *PDGFRα‐CreERT2* transgenic mice were crossed with *Tau‐mGFP* mice to generate *PLP‐CreERT* (either homozygous or heterozygous):*Tau‐mGFP* (heterozygous) or *PDGFRα‐CreERT2* (either homozygous or heterozygous):*Tau‐mGFP* (heterozygous) mice, respectively. Tamoxifen (Nacalai tesque, Kyoto, Japan) was dissolved in corn oil (Sigma, St. Louis, USA) to a final concentration of 10 mg/mL. *PLP‐CreERT:Tau‐mGFP* or *PDGFRα‐CreERT2:Tau‐mGFP* mice were injected intraperitoneally with 100 mg/kg tamoxifen at P8 for *PLP‐CreERT:Tau‐mGFP* mice, P15 or 6‐week‐old for *PDGFRα‐CreERT2:Tau‐mGFP* mice. All experiments were approved by the Institutional Animal Care and Use Committee and conducted per the guidelines for the care and use of animals at Jichi Medical University.

### 2.2 Injection of RV‐GFP Into the Cerebellum

For visualizing individual oligodendrocytes, we used RV‐GFP (Mori and Morimoto 2014). Briefly, 8‐week‐old C57BL/6 mice were anesthetized with an intraperitoneal injection of mixed anesthetic (0.3 mg/kg medetomidine, 4.0 mg/kg midazolam, and 0.5 mg/kg butorphanol) or anesthetized with isoflurane (2%–3%) and placed in a stereotaxic frame (Narishige, Tokyo, Japan). A solution of RV‐GFP (1 μL, 3.3 × 10^4^ IU) was stereotaxically injected into lobules IV/V of the cerebella (6.45 mm posterior and 1.00 mm lateral to the bregma, a depth of 0.5 mm). For P8 pups, anesthesia was induced using 3% isoflurane in a plastic chamber, followed by maintenance with 2.5% isoflurane during the stereotaxic surgery. RV‐GFP (0.5 μL, 1.7 × 10^4^ IU) was stereotaxically injected into the P8 cerebella (2.7 mm posterior and 0.44 mm lateral to the lambda, a depth of 0.35 mm), using pulled glass pipettes with an inner diameter of 20–40 μm and an air pressure system. The injection took approximately 3 min. The mice were sacrificed 4 days after the RV‐GFP injection.

### 2.3 Immunohistochemistry

Mice were perfused with 4% paraformaldehyde in 0.1 M phosphate buffer (PB) at the ages stated in each section. The 70 μm‐thick brain slices were prepared by a vibratome slicer (LinearSlicer PRO 10; DSK, Kyoto, Japan). Lobules IV/V of the cerebella were dissected from the brain slices using a liner paintbrush. The cerebellar slices were then postfixed in the same fixative for 24 h at 4°C. The slices were washed three times in PBS for 10 min each at room temperature (RT), and immunostaining was performed per the manufacturer's protocol of RapiClear 1.52 (RC152001, Sunjin Laboratory, Taiwan, China) with minor modifications. The slices were incubated in 2% PBST (2% Triton X‐100 and 0.05% sodium azide in PBS) for 24 h at RT. They were then washed three times with PBS for 10 min each and incubated in a blocking buffer (10% normal goat serum, 1% Triton X‐100, 2.5% DMSO, and 0.1% sodium azide in PBS) for 24 h at RT. The slices were further incubated with various antibodies, including chicken anti‐neurofilament‐H (1/500, NB300‐217, Novusbio, Colorado, USA), rabbit anti‐calbindin (1/500, MSFR100390, Frontier Institute, Hokkaido, Japan), rat anti‐GFP (1/500, 04404‐84, Nacalai tesque, Kyoto, Japan), anti‐APC (CC1, 1/100, OP80, Merk, Darmstadt, Germany), and rat anti‐MBP (1/100, MCA409S, Bio‐Rad, California, USA) in an antibody dilution buffer (1% normal goat serum, 0.2% Triton X‐100, 2.5% DMSO and 0.1% sodium azide in PBS) for 3–4 days at 4°C. The anti‐GFP antibody was used to visualize GFP in the *Tau‐mGFP* mice. They were then washed three times with a washing buffer (3% NaCl and 0.2% Triton X‐100 in PBS) for 1 h each at RT. The cerebellar slices were subsequently incubated with secondary antibodies in an antibody dilution buffer, including goat anti‐chicken IgY Alexa Fluor 647 (1/200, A‐21449, ThermoFisher, Massachusetts, USA), goat anti‐rat IgG Alexa Flour 488 (1/200, A‐11006, ThermoFisher) and goat anti‐rabbit IgG Alexa Flour 568 (1/200, A‐11011, ThermoFisher), for 2 days at 4°C. They were then washed three times with washing buffer for an hour each. The cerebellum slices were incubated with Hoechst 33342 (1/2000, H3570, ThermoFisher) for 2 h at RT. After a final wash with PBS for 10 min, the slices were placed on glass slides and each slice was treated with 15–20 μL of RapiClear solution for tissue clearing. Finally, the slices were mounted with RapiClear solution.

### 2.4 Confocal Microscopic Analysis

Series of Z‐stack projections were acquired at 0.5 μm intervals (objective lens ×60 or ×100; N.A., 1.42 or 1.45, respectively) using Dragonfly high speed confocal microscope system (Oxford instruments, Abingdon‐on‐Thames, UK) or FV1000 (objective lens ×60; N.A., 1.35) (Olympus, Tokyo, Japan). Analysis of individual oligodendrocytes and counting of axonal numbers were done using serial confocal microscopy images. We identified the top and bottom parts of each oligodendrocyte by detecting the disappearance of the RV‐GFP signal. We also determined the outermost processes using stacked serial images. Axons adjacent to the oligodendrocytes were defined as those falling within the range between the outermost processes, as calculated from the stacked serial confocal images. Following our previous study (Osanai et al. 2017), RV‐GFP (+) oligodendrocyte processes that are wrapping around axons labeled with anti‐calbindin and/or anti‐neurofilament antibodies were defined as myelin sheaths, and such myelin sheath was flanked by a paranodal marker, Contactin associated protein, in our analyses. The diameter of axons was calculated by measuring the full‐width at half maximum (FWHM) of fluorescence intensity using Fiji software. Briefly, five lines perpendicular to a targeted axon were drawn using Fiji, and histograms of fluorescence intensity for each line were made using the Plot profile function of Fiji. From the Gaussian fit of fluorescence intensity histogram, FWHM was calculated for each line as the distance between the two points on either side of the peak intensity where the fluorescent intensity falls to half of its maximum value. The average of FWHM of the five lines was calculated and determined as the diameter of a targeted axon.

### 2.5 Immunoelectron Microscopy Analysis

P12 C57BL/6 mice were anesthetized and perfused transcardially with saline, followed by a 4% paraformaldehyde and 2.5% glutaraldehyde solution in phosphate buffer. The brains were removed and postfixed overnight in the same fixative. Then, they were cut into 200‐μm‐thick sagittal slices using a vibratome slicer. Slices of the targeted region in the cerebellum (lobules 4/5) were gently dissected from the brain sections using a razor blade. The slices were washed with phosphate buffered saline (PBS) four times for 2 min each at 4°C, and then incubated in 5% bovine serum albumin (BSA) in PBS for 3 h at room temperature (RT). Subsequently, the cerebellar slices were incubated in rabbit anti‐calbindin (1/50, MSFR100390, Frontier Institute) diluted in PBS containing 5% BSA for 3 h at RT. The slices were then washed with PBS three times for 3 min each at RT. Next, the cerebellar slices were incubated in an HRP‐conjugated anti‐rabbit IgG (1:100, 111‐035‐144; Jackson ImmunoResearch, Pennsylvania, USA) for 1 h at RT and washed with PBS three times for 3 min each at RT. Finally, the cerebellar slices were incubated in DAB solution (0.5 mg/mL DAB with 10 nM H_2_O_2_ in PBS) for 10 min, and then washed with PBS three times for 3 min each at RT. Thereafter, the samples were stained en bloc, dehydrated and embedded in resin, as described previously (Morizawa et al. 2022). Briefly, slices were washed four times in ice cold PBS and then treated with PBS containing 2% OsO_4_ (Nisshin EM, Tokyo, Japan) and 1.5% K[Fe(CN)_6_] (Nacalai tesque) on ice. The slices were then incubated with 1% thiocarbohydrazide (Tokyo Chemical Industry, Tokyo, Japan) for 20 min and then with 2% OsO4 for 30 min at RT. The slices were treated with 2% uranyl acetate at 4°C overnight and for an additional 30 min at RT. They were then stained with Walton's lead aspartate at 50°C for 2 h. Each of these treatments was followed by washing four times in double distilled water. The slices were then dehydrated with a graded ethanol series (60%–80%–90%–95%) at 4°C, infiltrated sequentially with dehydrated acetone, a 1:1 mixture of acetone and Durcupan (Sigma), a 1:3 mixture of acetone and Durcupan, and finally 100% Durcupan. The incubation in Durcupan was done overnight and then repeated in fresh Durcupan for an additional 90 min. Then Durcupan was polymerized at 60°C for 3 days. The pieces of Durcupan with samples were attached on aluminum rivets, trimmed and imaged with a field emission type scanning electron microscope equipped with the 3View system and the OnPoint backscattered electron detector (Gatan, Pleasanton, CA). Serial images were acquired at the resolution of 5.0 nm/pixel and 50 nm steps in the depth direction. The images were processed using Fiji (https://fiji.sc/) and Amira (ThermoFisher).

### 2.6 Statistical Analysis

All statistical analyses were performed using Prism 7 (GraphPad Software). Using confocal images with GFP‐labeled oligodendrocytes and their processes and myelin sheaths, calbindin (+) and (−) axons were identified, and the numbers of calbindin (+) and (−) axons myelinated and not myelinated by GFP‐labeled myelin were counted. Fisher's exact test was used to assess preference toward calbindin (+) or (−) axons. The one‐way ANOVA with Dunn's multiple comparisons was used for nonparametric multiple comparisons. The boxes and bars in the graphs indicate the mean ± SD.

## 3 Results

### 3.1 Oligodendrocytes Preferentially Myelinate Purkinje Cell Axons in the Cerebellar White Matter of Adult Mice

**FIGURE 1:**
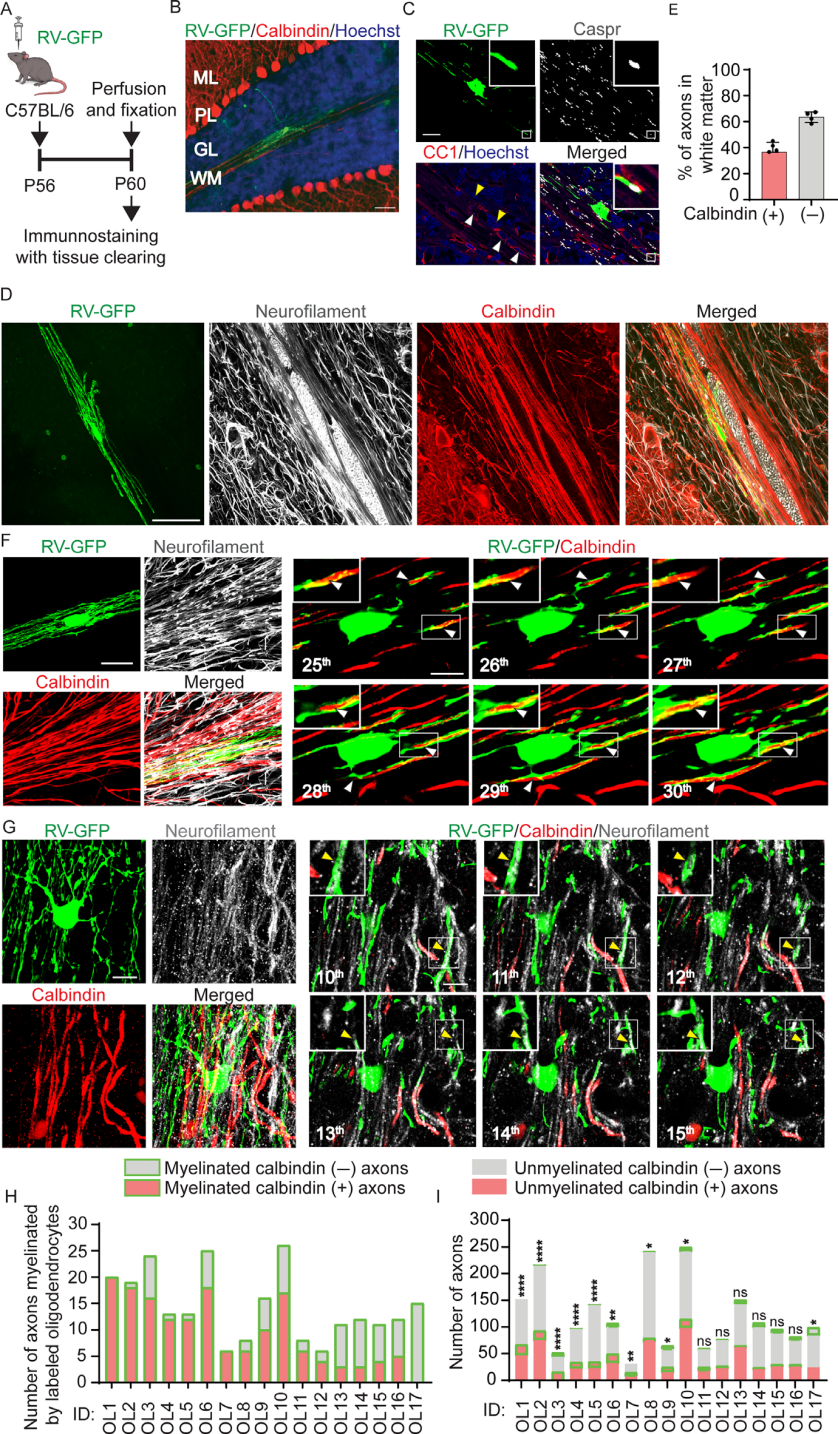
Oligodendrocytes preferentially myelinate Purkinje cell axons in the cerebellar white matter of adult mice. (A) Methods and experimental timeline of injection of the rabies virus encoding GFP (RV‐GFP) and sample preparation. (B) A representative image showing an oligodendrocyte sparsely labeled with RV‐GFP (green) in the cerebellar white matter. Purkinje cells are immunostained for calbindin (red), and nuclei are stained by Hoechst (blue). Scale bar: 50 μm. GL, granular layer; ML, molecular layer; PL, Purkinje cell layer; WM, white matter. (C) RV‐GFP‐labeled oligodendrocytes (green) with immunostaining for a marker of mature oligodendrocyte, CC1 (red), and a paranodal marker, contactin‐associated protein (Caspr, white) and nuclei stained with Hoechst (blue). The area marked with a rectangle is magnified in the insets. Yellow arrowheads indicate CC1‐negative cells, while white arrowheads indicate CC1‐positive cells. Scale bars: 20 μm. (D) Representative images of an RV‐GFP‐labeled oligodendrocyte (green) and axons immunostained for neurofilament (white) and calbindin (red). Scale bar: 30 μm. (E) The percentage of calbindin‐positive/neurofilament‐positive (calbindin (+)) and calbindin‐negative/neurofilament‐positive (calbindin (−)) axons in the cerebellar white matter. (F) The left panel shows Z‐stacked confocal microscopy images of calbindin (+) and (−) axons and an RV‐GFP‐labeled oligodendrocyte which preferentially myelinates calbindin (+) axons. The right panel shows serial confocal microscopy images of the left panel, where the oligodendrocyte myelinates calbindin (+) axons (white arrowheads). The area marked with a rectangle is magnified in insets. Scale bars: 20 μm (left) or 10 μm (right). (G) The left panel shows Z‐stacked confocal microscopy images of calbindin (+) and (−) axons and an RV‐GFP‐labeled oligodendrocyte preferentially myelinating calbindin (−) axons. The right panel shows serial confocal microscopy images of the left panel, where the oligodendrocyte myelinates calbindin (−) axons (yellow arrowheads). The area marked with a rectangle is magnified in insets. Scale bars: 20 μm (left) or 10 μm (right). (H) The number of calbindin (+) and calbindin (−) axons myelinated by the single RV‐GFP‐labeled oligodendrocytes. Each oligodendrocyte is shown using an ID number (OL1–OL17). Data were obtained from 17 oligodendrocytes from four mice. (I) The numbers of calbindin (+) and (−) axons myelinated by each RV‐GFP‐labeled oligodendrocyte (Myelinated calbindin (+) and (−) axons, respectively) and neighboring calbindin (+) and (−) axons not myelinated by the oligodendrocyte (Unmyelinated calbindin (+) and (−) axons, respectively). *p*‐values of Fisher's exact test are shown on the bars. ****p < 0.0001, **p < 0.01, *p < 0.05, ns: *p* > 0.05. The same ID numbers represent the same oligodendrocytes (G, H).

To identify the neuronal subtypes myelinated by single oligodendrocytes, we sparsely labeled oligodendrocytes in the cerebellar white matter of 8‐week‐old mice using attenuated rabies virus encoding GFP (RV‐GFP) (Osanai et al. 2017). Subsequently, immunohistochemistry (IHC) was performed along with a tissue clearing to identify the axonal subtypes (Figure 1A–D). GFP‐labeled cells with mature oligodendrocyte‐like morphology were confirmed to be positive for the mature oligodendrocyte marker CC1, with their processes flanked by the paranodal marker contactin‐associated protein (Caspr), confirming that the GFP‐labeled cells were myelinating oligodendrocytes (Figure 1C). Using anti‐calbindin and anti‐neurofilament antibodies, we distinguished Purkinje cell axons (calbindin (+)/neurofilament (+); hereafter, calbindin (+) axons) and other axons, including mossy fibers and climbing fibers (calbindin (−)/neurofilament (+) axons; hereafter, calbindin (−) axons) (Figure 1D). In general, the number of calbindin (+) axons was lower than that of calbindin (−) axons (calbindin (+) versus (−): 36.65% ± 4.06% versus 63.45% ± 4.06%) (Figure 1E). Using a combination of RV‐GFP and IHC with tissue clearing, we observed that RV‐GFP‐labeled oligodendrocytes extended processes and myelinated calbindin (+) or (−) axons (Figure 1F,G). We counted the calbindin (+) and (−) axons myelinated by RV‐GFP‐labeled oligodendrocytes (Figure 1H), as well as those that passed near the labeled oligodendrocytes but were not myelinated by them (Figure 1I). The processes that ensheathed axons for more than 20 μm were defined as the myelin sheaths (Osanai et al. 2017). We used Fisher's exact test to assess bias toward calbindin (+) or (−) axons in each oligodendrocyte and found that eight out of 17 (about 47%) GFP‐labeled oligodendrocytes preferentially myelinated calbindin (+) axons (Figure 1I), while one oligodendrocyte preferentially myelinated calbindin (−) axons (Figure 1I). These results indicate that substantial proportion of oligodendrocytes preferentially myelinate calbindin (+) axons in the cerebellar white matter of adult mice.

### 3.2 Oligodendrocytes Preferentially Myelinate Purkinje Cell Axons in the Early Developmental Period

Since the axonal diameter regulates the initiation of myelination and a single oligodendrocyte tends to myelinate axons with a discrete range of diameters, we analyzed the diameters of calbindin (+) and (−) axons (Call and Bergles 2021; Lee et al. 2013; Madisen et al. 2015; Osso, Rankin, and Chan 2021; Stedehouder et al. 2019; Tanaka et al. 2021; Uchida et al. 2012). We estimated the diameter of calbindin (+) and (−) axons, both myelinated and unmyelinated by the GFP‐labeled oligodendrocytes, by measuring the full‐width at half maximum (FWHM) of the fluorescent axonal profiles (Osso, Rankin, and Chan 2021; Stedehouder et al. 2019). FWHM is defined as the distance between the two points on either side of the peak intensity where the fluorescence intensity falls to half of its maximum value. There was no significant diameter difference between axons myelinated and those not myelinated by GFP‐labeled oligodendrocytes (Figure S1). These results suggested that myelination preference toward calbindin (+) axons is not caused by a bias for larger axonal diameter.

**FIGURE 2:**
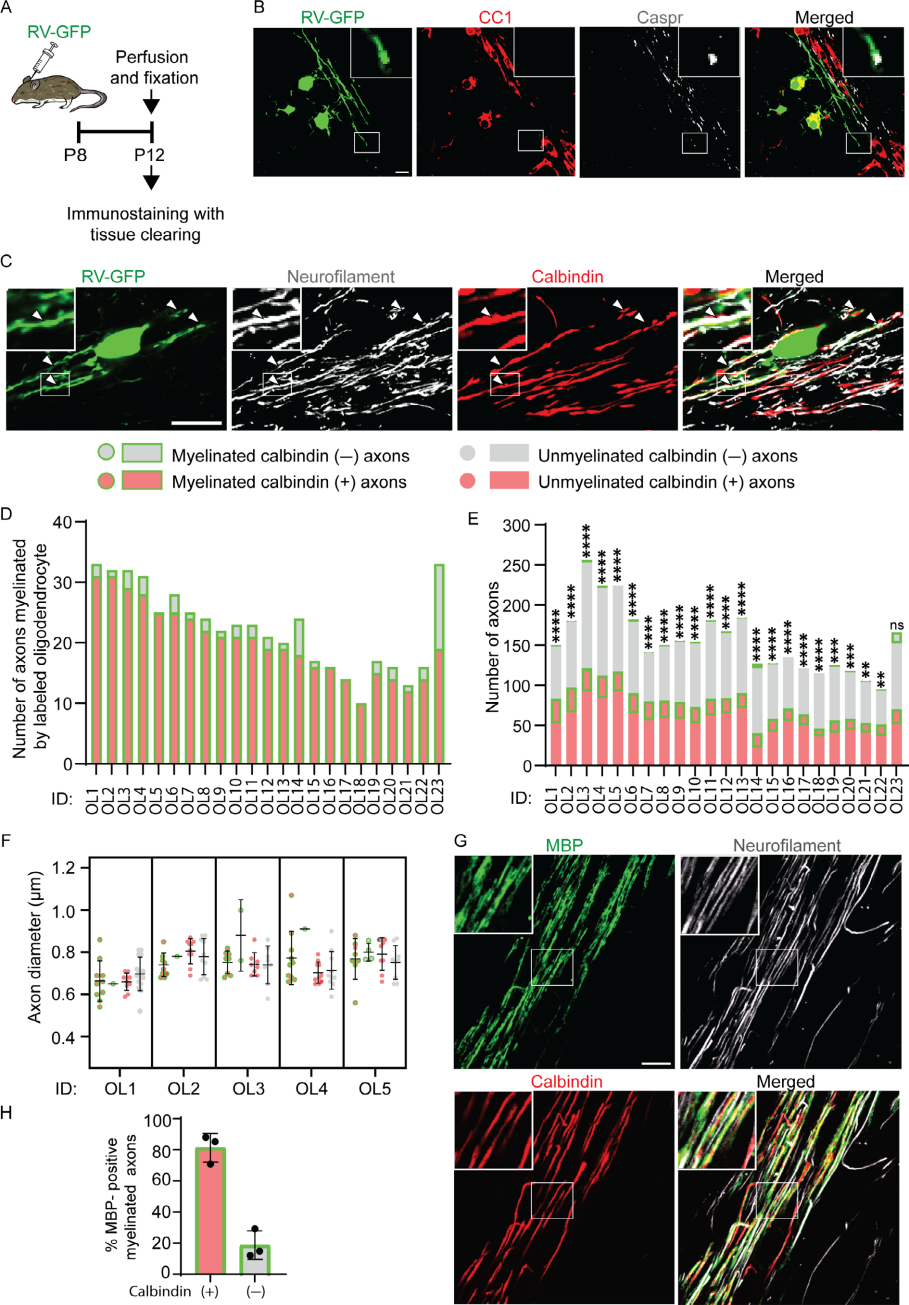
Oligodendrocytes preferentially myelinate Purkinje cell axons at early development. (A) Method and experimental timeline of rabies virus encoding GFP (RV‐GFP) injection and sample preparation. (B) RV‐GFP‐labeled oligodendrocytes (green) with immunostaining for a marker of mature oligodendrocyte, CC1 (red), and a paranodal marker, contactin‐associated protein (Caspr, white). The area marked with a rectangle is magnified in insets. Scale bar: 15 μm. (C) Representative fluorescence images of P12 mouse cerebellar white matter with an RV‐GFP‐labeled oligodendrocyte and immunostaining for calbindin and neurofilaments, showing GFP‐positive myelination of calbindin‐positive (calbindin (+)) axons (arrowheads). The area marked with a rectangle is magnified in insets. Scale bar: 20 μm. (D) The number of calbindin (+) and calbindin‐negative (calbindin (−)) axons myelinated by the single RV‐GFP‐labeled oligodendrocyte. Each oligodendrocyte is shown using an ID number (OL1–OL23). (E) The numbers of calbindin (+) and (−) axons myelinated by each of RV‐GFP‐labeled oligodendrocyte (Myelinated calbindin (+) and (−) axons, respectively) and neighboring calbindin (+) and (−) axons not myelinated by the oligodendrocyte (Unmyelinated calbindin (+) and (−) axons, respectively). *p*‐values of Fisher's exact test are shown on the bars. ****p < 0.0001, ***p < 0.001, **p < 0.01, ns: *p* > 0.05. The same ID numbers represent the same oligodendrocytes (D, E). The data were obtained from 23 oligodendrocytes in five mice. (F) Diameters of calbindin (+) and (−) axons that are myelinated or unmyelinated by the RV‐GFP‐labeled oligodendrocytes. Each dot represents one axon and bars show the mean ± SD. (G) Immunostaining for a myelin marker, myelin basic protein (MBP, green), calbindin (red), and neurofilament (white) in the cerebellar white matter at P12. The area marked with a rectangle is magnified in insets. Scale bar: 20 μm. (H) Percentages of calbindin (+) and (−) axons in total axons myelinated by MBP immunostaining.

It is known that myelination of the mouse cerebellum begins around P5 to P8 (Chiba et al. 2016; Groteklaes et al. 2020). To analyze the myelination preference at the early stage of cerebellar myelination, RV‐GFP was injected into the white matter of P8 mouse cerebellum, and the mice were sacrificed at P12 to identify the axonal subtypes myelinated by the labeled oligodendrocytes (Figure 2A). In the cerebellar white matter, GFP‐labeled oligodendrocytes tested positive for a mature oligodendrocyte marker, CC1, and their processes formed myelin sheaths flanked by a paranodal marker, Contactin‐associated protein (Caspr, Figure 2B). We found that 22 out of 23 oligodendrocytes (approximately 96%) preferentially myelinated the calbindin (+) axons (Figure 2C–E). The diameters of myelinated calbindin (+) and (−) axons were similar at P12 (Figure 2F). If most early‐born oligodendrocytes predominantly myelinate calbindin (+) axons, in general, the myelin sheath should be predominantly formed on calbindin (+) axons at the beginning of cerebellar myelination. When percentages of myelinated calbindin (+) and calbindin (−) axons were analyzed using immunostaining for a myelin marker, myelin basic protein (MBP), we found that 81.34% ± 9.2% of myelinated axons were calbindin (+) axons in the cerebellar white matter at P12 (Figure 2G,H). Taken together, these data indicate that early‐born oligodendrocytes selectively myelinate calbindin (+) axons, which are the major axonal subtype initially myelinated in the cerebellar white matter.

**FIGURE 3:**
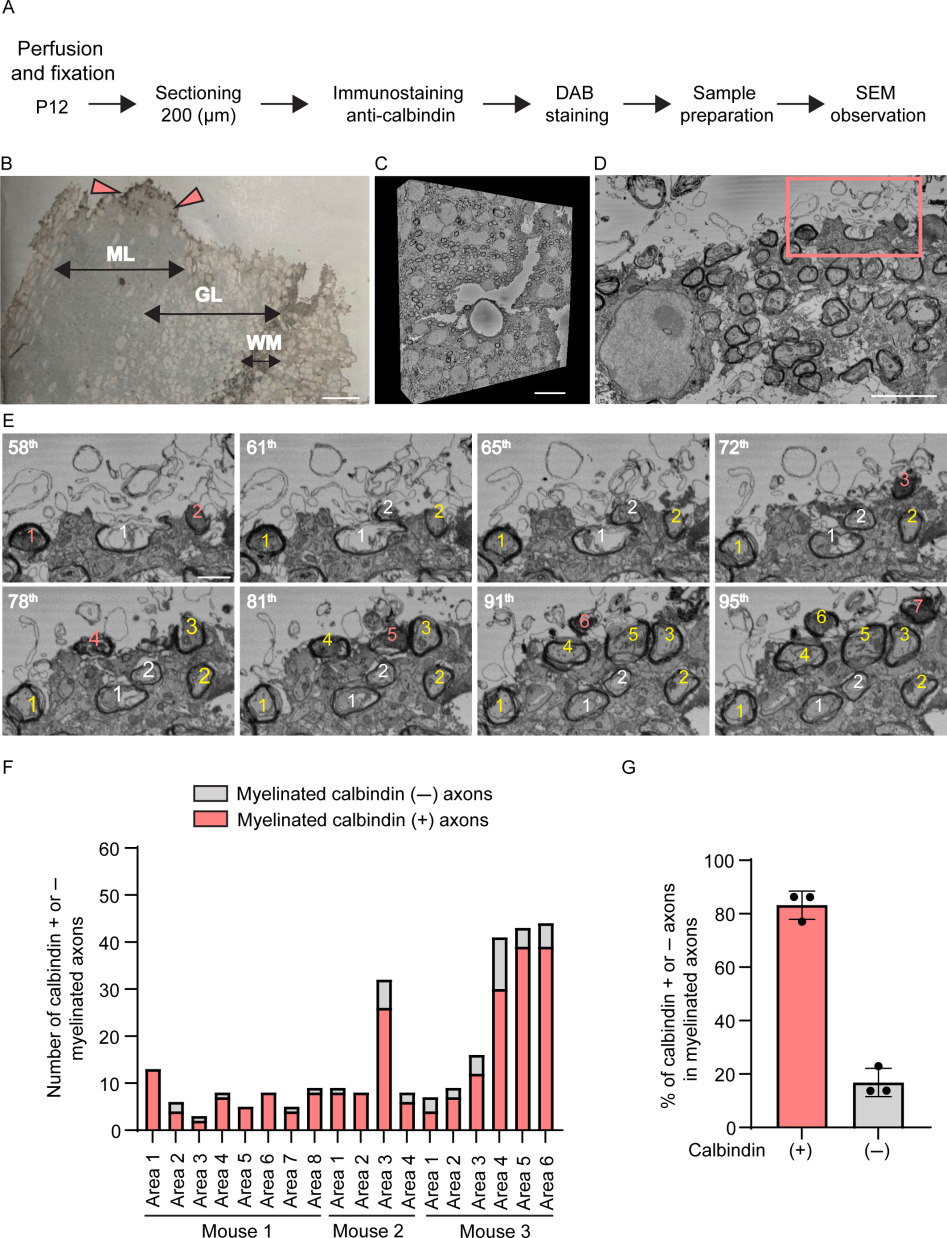
Predominant compact myelin formation around Purkinje cell axons in the cerebellar white matter of P12 mice. (A) An experimental protocol of serial immunoelectron microscopy with serial block‐face scanning electron microscopy. DAB, diaminobenzidine. (B) A light microscopic image of a tissue slice immunostained for calbindin with DAB visualization and embedded in epoxy resin. Myelinated axons are observed in cerebellar white matter (WM) and only the tissue surface has DAB signals (arrowheads). GL, granular layer; ML, molecular layer. Scale bar: 20 μm. (C) Three‐dimensional reconstruction of the serial immunoelectron microscopic images acquired from the area including cerebellar white matter. Scale bar: 10 μm. (D) One of the serial images of cerebellar white matter including the area indicated with a rectangle and magnified in (E). Scale bar: 2 μm. (E) Serial images at a higher magnification showing DAB signals corresponding to calbindin immunoreactivity in myelinated axons near the tissue surface. The slice numbers in the image stack are shown in the upper left corners. Myelinated calbindin (+) and calbindin (−) axons are numbered with yellow and white numbers, respectively. The axonal numbers are colored red when DAB signals can be detected in the image. The calbindin (+) axons show DAB signals in the images near the surface of the tissue; however, the DAB signal of the profile fades in the deeper areas of the tissue. Scale bar: 500 nm. (F) Numbers of myelinated calbindin (+) and calbindin (−) axons in each of the observed areas from different mice. Calbindin (+) and (−) axons are represented by red and gray bars, respectively. (G) Percentage of myelinated calbindin (+) and calbindin (−) axons. Each dot represents a mouse.

To further confirm that myelin sheaths were dominantly formed on calbindin (+) axons at the early stage of developmental myelination, we performed serial immunoelectron microscopic analyses in the cerebellar white matter of P12 mice. The fixed cerebellar slices were immunostained for calbindin which was visualized with diaminobenzidine (DAB), embedded in resin following *en bloc* staining and observed with serial block‐face scanning electron microscopy (Figure 3A). Cerebellar white matter was identified in the immunostained tissue block (Figure 3B), and in the serial electron microscopic images (Figure 3C), myelin sheaths were identified around some axons (Figure 3D). Some cells observed around myelin sheaths might be oligodendrocytes forming the sheaths; however, tracing their processes for confirmation was not possible in this study. Since treatments that destroy tissue structures and increase antibody penetration were minimized, the DAB signal was present only near the surface of the tissue (Katoh et al. 2017). Therefore, the calbindin immunopositive DAB signals were identified near the surface of the tissues, and we tracked the profiles of myelinated axons that tested positive or negative for the DAB signals in the serial images (Figure 3E). Consequently, we found that the majority of the myelinated axons in the cerebellar white matter were calbindin (+) at this age (Figure 3F). Consistent with the immunofluorescence results (Figure 2H), 83.19% ± 5.3% of myelinated axons were calbindin (+) axons (Figure 3G). Taken together, these data indicate that oligodendrocytes that matured before P12 preferentially myelinate calbindin (+) axons.

### 3.3 Early Differentiated Oligodendrocytes Remain to Preferentially Myelinate Purkinje Cell Axons in Adult Mouse Cerebella

**FIGURE 4:**
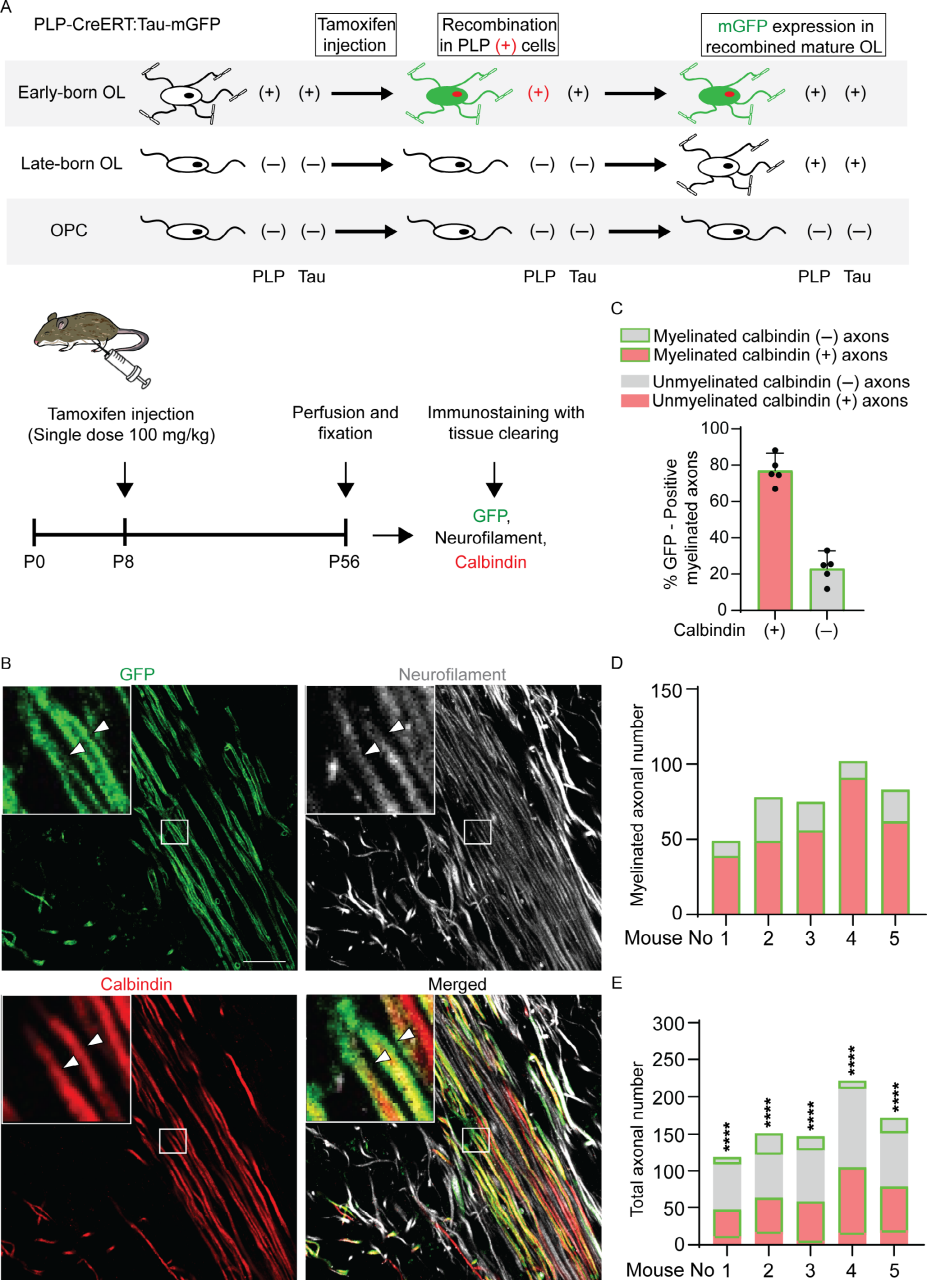
Early differentiated oligodendrocytes remain to preferentially myelinate calbindin‐positive axons in adult mouse cerebella. (A) Schematic diagram of the experimental design using *PLP‐CreERT: Tau‐mGFP* mice. OL, oligodendrocyte; OPC, oligodendrocyte precursor cell; PLP, proteolipid protein. In *PLP‐CreERT:Tau‐mGFP* mice, the mature OLs that were differentiated and became *PLP*‐positive before tamoxifen injection (Early‐born OL) are labeled with GFP, while those differentiated after the injection (Late‐born OL) or OPC are not labeled. (B) Representative immunofluorescence images of a *PLP‐CreERT: Tau‐mGFP* transgenic mouse brain section. A marked area in the stacked image is magnified in the insets. Myelin sheaths of GFP‐labeled oligodendrocytes (green) cover calbindin‐positive (calbindin (+), arrowheads) and calbindin‐negative (calbindin (−)) axons. Scale bar: 20 μm. (C) The percentage of calbindin (+) and (−) axons myelinated by GFP‐labeled myelin in the cerebellar white matter. Each dot represents one mouse. (D, E) The numbers of calbindin (+) and (−) axons myelinated by GFP‐labeled myelin in each mouse (D, E, Myelinated calbindin (+) and (−) axons, respectively) and neighboring calbindin (+) and (−) axons not myelinated by GFP‐labeled myelin (E, Unmyelinated calbindin (+) and (−) axons, respectively) in each mouse. *p*‐values of Fisher's exact test are shown on the bars. ****p < 0.0001. The same mouse ID numbers represent the same mice.

Since oligodendrocytes can dynamically form new myelin sheaths in addition to the initially formed ones (Duncan et al. 2018; Jeffries et al. 2016; Mezydlo et al. 2023), it remains unclear whether early‐born oligodendrocytes continue to preferentially myelinate Purkinje cell axons in adult animals. To determine if the preferential myelination of early differentiated oligodendrocytes toward calbindin (+) axons that remain in the adult cerebellar white matter, we used *PLP‐CreERT: Tau‐lox‐STOP‐lox‐mGFP* transgenic mice (hereafter *PLP‐CreERT: Tau‐mGFP*). In these mice, membrane‐targeted GFP is expressed only in the mature oligodendrocytes with PLP expression at the time of tamoxifen exposure, while those that matured afterward are not labeled with GFP (Figure 4A). To label early‐born oligodendrocytes, tamoxifen was administered at P8 and the mice were sacrificed at P56. In these analyses, it was not possible to identify individual GFP‐labeled oligodendrocytes because they were labeled too densely. However, axonal subtypes myelinated by GFP‐positive myelin were identified and distinguished from those not myelinated by the GFP‐positive myelin (Figure 4B). We found that GFP‐labeled myelin sheaths were dominantly formed on calbindin (+) axons even in the P56 mouse cerebella (Figure 4C). In the axons myelinated by the GFP‐labeled myelin sheaths in each mouse, the calbindin (+) axons outnumbered the calbindin (−) ones (Figure 4D). The results of Fisher's exact test for analyzing myelination bias toward calbindin (+) or calbindin (−) axons showed that GFP‐labeled myelin sheaths dominantly formed on the calbindin (+) axons in each mouse (Figure 4E). These results indicate that early‐born oligodendrocytes preferentially myelinate calbindin (+) axons in the cerebellar white matter of adult mice.

### 3.4 Later‐Differentiated Oligodendrocytes Preferentially Myelinate Calbindin (−) Axons

**FIGURE 5:**
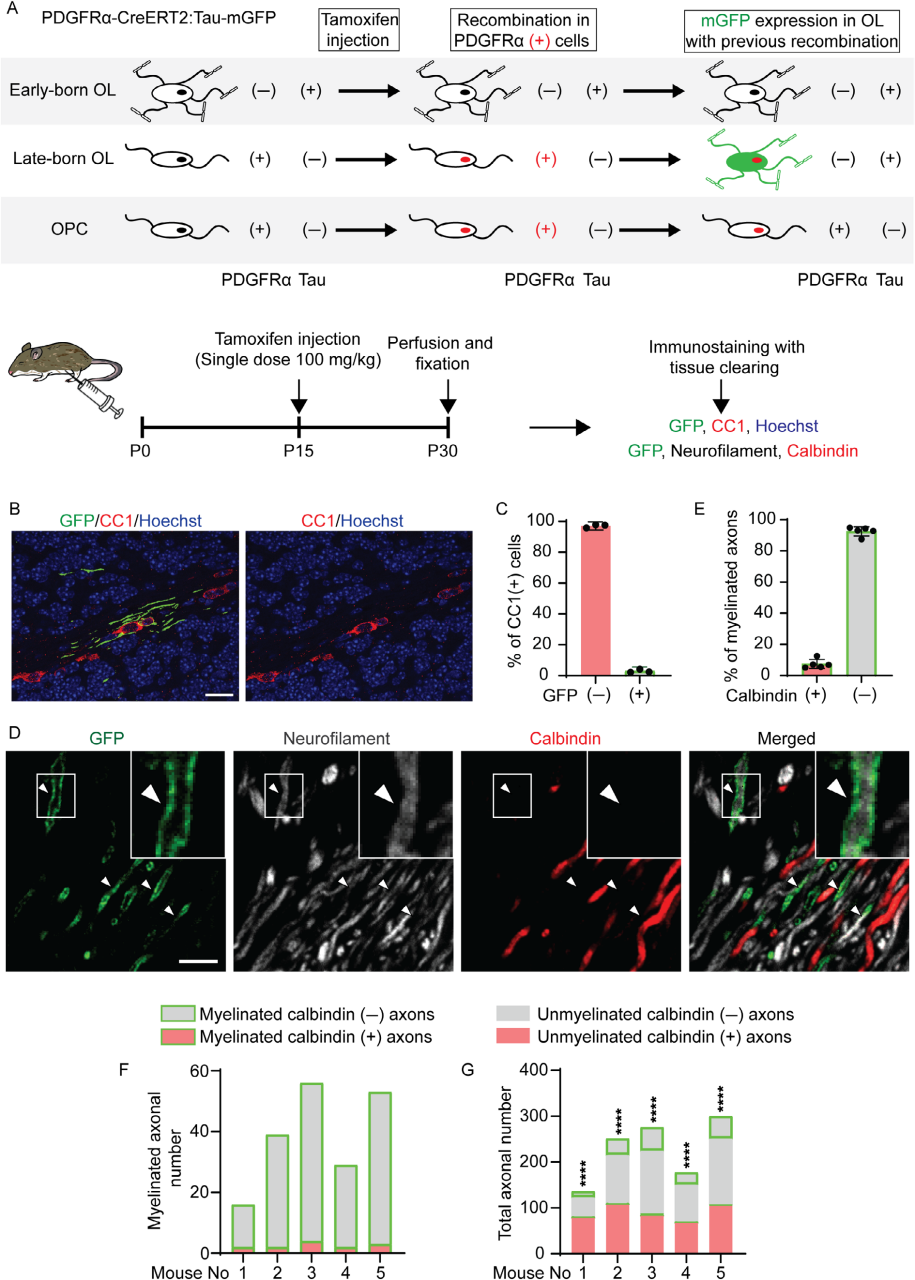
Later‐differentiated oligodendrocytes preferentially myelinate calbindin (−) axons. (A) Schematic diagram of the experimental design using *PDGFRα‐CreERT2:Tau‐mGFP* mice. OL, oligodendrocyte; OPC, oligodendrocyte precursor cell; PDGFRα, platelet‐derived growth factor receptor alpha, an OPC marker. In *PDGFRα‐CreERT2: Tau‐mGFP* mice, the Tau‐positive (Tau (+)) mature OL differentiated after the tamoxifen injection (Late‐born OL) are labeled with GFP, while OL differentiated before the injection (Early‐born OL) or OPC are not labeled. (B) A fluorescence image of GFP‐labeled oligodendrocytes (green) with fluorescent staining for a marker of mature oligodendrocytes, CC1 (red), and nuclei (Hoechst, blue). Scale bar: 10 μm. (C) The percentage of GFP‐labeled CC1‐positive mature oligodendrocytes among all CC1‐positive mature oligodendrocytes (2.96 ± 1.07%). (D) Representative immunofluorescence images of a P30 *PDGFRα‐CreERT2:Tau‐mGFP* mouse brain section with tamoxifen injection at P15. Myelin sheaths of GFP‐labeled oligodendrocytes (green) cover calbindin (−) axons (arrowheads). The area marked with a rectangle is magnified in insets. Scale bar: 5 μm. (E) The percentage of myelinated calbindin (−) and calbindin (+) axons among axons myelinated by GFP‐labeled myelin sheathes. (F, G) The numbers of calbindin (+) and (−) axons myelinated by GFP‐labeled myelin in each mouse (F, G, Myelinated calbindin (+) and (−) axons, respectively) and neighboring calbindin (+) and (−) axons not myelinated by GFP‐labeled myelin (G, Unmyelinated calbindin (+) and (−) axons) in each mouse. *p*‐values of Fisher's exact test are shown on the bars. ****p < 0.0001. The same mouse ID numbers represent the same mice.

Almost all axons in the cerebellar white matter of the adult mouse cerebellum are myelinated, and a fraction of oligodendrocytes showed a preference for calbindin (−) axons (Figure 1) (Nguyen et al. 2018). If the preference toward calbindin (+) and (−) is dependent on developmental timing, it is possible that later‐differentiated oligodendrocytes dominantly myelinate calbindin (−) axons. To analyze the preference of myelination in later‐differentiated oligodendrocytes, we used *PDGFRα‐CreERT2:Tau‐lox‐STOP‐lox‐mGFP* (hereafter *PDGFRα‐CreERT2:Tau‐mGFP*) transgenic mice. In the transgenic mice, the *lox‐STOP‐lox* cassette is removed in *PDGFRa* (+) OPC by tamoxifen administration and GFP will be expressed when the recombined cells become *Tau* (+) mature oligodendrocytes (Figure 5A). In other words, mature oligodendrocytes that differentiated after tamoxifen injection will be labeled with GFP (Young et al. 2013). To label late‐born oligodendrocytes, tamoxifen was administered at the age of 8 weeks or P15, and the mice were sacrificed at the age of 10 weeks or P30 (Figure 5A). There were few GFP‐positive oligodendrocytes in the cerebellar white matter at the age of 10 weeks in the mice that were administered tamoxifen at the age of 8 weeks (only one oligodendrocyte in the cerebellar white matter of five mice), suggesting that oligodendrocyte differentiation in cerebellar white matter is almost completed by the age of 8 weeks. In the cerebella of mice that were administered tamoxifen at P15, only a few CC1‐positive mature oligodendrocytes were labeled by GFP (3% of the total CC1‐positive oligodendrocytes, Figure 5B), suggesting that of the majority of oligodendrocytes were differentiated before being labeled with tamoxifen injection at P15 (Figure 5B,C). Then, we analyzed myelination toward calbindin (+) and (−) axons by the oligodendrocytes labeled with tamoxifen injection at P15 and found that the axons myelinated by these oligodendrocytes are mostly calbindin (−) at P30 (92.53 ± 2.937% of axons with GFP‐positive myelin sheaths) (Figure 5D,E). Nearby calbindin (+) axons did not outnumber calbindin (−) ones, and the results of Fisher's exact test for analyzing myelination bias toward calbindin (+) or calbindin (−) axons showed that GFP‐labeled myelin sheaths dominantly formed on the calbindin (−) axons in each mouse (Figure 5F,G). These results indicate that the later‐born oligodendrocytes predominantly myelinate calbindin (−) axons in the cerebellar white matter of adult mice.

## 4 Discussion

In this study, we combined labeling of single oligodendrocytes with an attenuated rabies virus vector and immunostaining with a tissue clearing technique and found that a certain population of oligodendrocytes preferentially myelinate calbindin (+) Purkinje cell axons in the cerebellar white matter. We also demonstrated that, in the adult cerebellar white matter, the oligodendrocytes that differentiated in the early developmental stage predominantly myelinate Purkinje cell axons, while those that differentiated later preferentially myelinate non‐Purkinje cell axons. To the best of our knowledge, this is the first report to demonstrate axonal selectivity in myelination by individual oligodendrocytes in the cerebellum and explain how selective myelination observed in adulthood is established.

The combination of the sparse oligodendrocyte labeling and immunohistochemical classification specific axons enabled thorough examinations of axonal preferences of each oligodendrocyte and stringent statistical evaluation. Structural analyses of individual oligodendrocytes (including their myelin sheaths) have been difficult in the white matter, where many axons and oligodendrocytes are densely distributed. However, sparse labeling of oligodendrocytes allowed us to visualize morphology of a single oligodendrocyte (Osanai et al. 2017). In order to observe interaction between single oligodendrocytes and neuronal axons, we previously used AAVs to label a specific subtype of axons. However, the efficiency of axonal labeling with AAV was low, and statistical comparisons of the preference to specific axons were difficult because the proportion of unlabeled axons is unknown. By contrast, immunohistochemical labeling visualizes most axons; therefore, it was possible to classify axons with specific markers and identify axons that are myelinated or not myelinated by the GFP‐labeled oligodendrocytes. In addition, the rigorous statistical evaluation can provide dependable results regarding axon preference. Previous studies using 3D‐EM analysis observed myelination by single oligodendrocytes toward specific neuron subtypes (Tanaka et al. 2021; Zonouzi et al. 2019). In this study, attenuated rabies virus and immunostaining allowed high‐throughput analysis of neuron subtype‐specific myelination. The method developed in this study will apply to other CNS regions, especially white matter with large caliber axons, and provide detailed maps of axonal preference in myelination by individual oligodendrocytes.

A clear preference for Purkinje cell axons was observed in early‐born oligodendrocytes. Previous studies reported that oligodendrocytes can wrap myelin around cylindrical or fibrous materials, proposing the concept that oligodendrocytes are unselective and use physical cues for myelination (Almeida 2018; Lee et al. 2012). However, myelination must be finely adjusted for axons within neuronal networks, including elaborate spatial organization and structural modulation of myelin to precisely control the arrival timings of action potentials (Almeida 2018; Almeida and Lyons 2017; Richardson, McIntyre, and Grill 2000; Yamazaki et al. 2014, 2019). Recent studies have suggested that cell adhesion molecules, which are expressed in myelin‐forming cells and neurons, can specifically interact with one another and facilitate or repel myelination (Djannatian et al. 2019; Elazar et al. 2019; Sukhanov et al. 2021). One possibility is that oligodendrocytes selectively myelinate Purkinje cell axons depending on N‐cadherin expression. Previous studies have shown that inhibiting N‐cadherin function results in delayed myelination or hypomyelination of neuronal axons, including Purkinje cell axons (Chen et al. 2017; Lewallen et al. 2011; Schnädelbach et al. 2001). N‐cadherin or other cell adhesion molecules may be expressed in Purkinje cell axons during early development, leading to Purkinje cell‐specific myelination. Conversely, non‐Purkinje cell axons may express repulsive molecules, such as JAM2, to inhibit myelination during early development (Redmond et al. 2016). Myelination has been suggested that to limit neuronal plasticity (McGee et al. 2005; Xin et al. 2024); therefore, immature non‐Purkinje cell axons may avoid myelination to maintain neuronal plasticity. GABAergic signaling has been shown to modulate oligodendrocyte maturation (Hamilton et al. 2017; Zonouzi et al. 2015), and some oligodendrocytes preferentially myelinate GABAergic neurons in the cerebral cortex (Zonouzi et al. 2019). Thus, GABA expression from Purkinje cells may contribute to Purkinje cell‐specific myelination. Identifying genes responsible for this specific myelination could be achieved by comparing gene expression between Purkinje and non‐Purkinje cells at early and later developmental stages. Uncovering the mechanisms of selective myelination may ultimately lead to the discovery of genes that promote myelination. Further studies are required to clarify the mechanisms of selective myelination.

The preference toward Purkinje cell axons was more prominent immediately after myelination was initiated. The finding that early‐born oligodendrocytes preferentially myelinate a particular subtype of neuronal axons is consistent with the developing zebrafish spinal cord, where Mauthner axons are the first to be myelinated (Almeida et al. 2011). Mauthner cells and Purkinje cells share the characteristics of being large neurons. It is possible that oligodendrocytes preferentially myelinate the axons of these large neurons to support the high metabolic demands of their large cell bodies and extensive axonal processes. Additionally, these neurons may mature earlier than others, leading to early myelination due to their importance in survival function such as escape responses and motor control.

In certain hereditary leukodystrophies, cerebellar atrophy and cerebellar ataxia are observed. In Pelizaeus–Merzbacher disease, cerebellar symptoms such as nystagmus and intention tremor are manifest in infants. These symptoms may be attributed to the hypomyelination of Purkinje cell axons, which are selectively myelinated during the early developmental period. In addition, white matter damage is commonly observed in neonatal hypoxic–ischemic encephalopathy (Martinez‐Biarge et al. 2012). It is possible that Purkinje cell axon myelination is predominantly affected by neonatal hypoxic–ischemic encephalopathy. Neonatal hypoxic–ischemic encephalopathy can cause motor and cognitive dysfunction in survivors (Lee and Glass 2021). Because the Purkinje cells are principal neurons that project from the cerebellar cortex, Purkinje cell axons are responsible for major cerebellar functions such as motor coordination, and damage of the myelin sheaths on Purkinje cell axons will significantly affect cerebellar function. It is likely that Purkinje cell selective myelination is necessary for eye movement and motor coordination as these functions are significantly affected in leukodystrophy of infant. Revealing the functional consequence of Purkinje cell‐specific myelination and its interruption will be important for understanding the pathogenesis of neonatal hypoxic–ischemic encephalopathy and other cerebellar diseases.

The outputs through functionally different axons such as Purkinje cell axons and climbing fibers may require the differential regulation of axonal conduction and important targets of selective myelination. In addition, the maintenance of preferential myelination by early differentiating oligodendrocytes until adulthood raises the hypothesis that the preference observed in adulthood is regulated by the timing of myelination during development; early‐ and late‐myelinating oligodendrocytes may determine neural circuit regulation through preferential myelination. If so, any developmental disturbance and/or event affecting myelination would affect the normal patterns of axonal selectivity and modulate the roles of oligodendrocyte myelination in conduction regulation and the group of axons affected by oligodendrocyte abnormality in adulthood. Further studies are required to reveal the functional consequences of preferential myelination toward a particular class of axon.

## Author Contributions

B.B. and Y.O. conducted experiments and acquired the data. Y.O. and N.O. conceptualized and designed the study. R.Y. and Y.S. assisted with methodologies. N.O. supervised the project. B.B., Y.O., and N.O. wrote the paper. All authors critically reviewed and approved the manuscript.

## Ethics Statement

This study has been approved by Institutional Animal Care and Use Committee and Committee for Genetic Recombination Experiments of Jichi Medical University.

## Conflicts of Interest

The authors declare no conflicts of interest.

## Supporting information


Figure S1.

## Data Availability

The data that support the findings of this study are available from the corresponding author upon reasonable request.
