# Supplementary material for: Axonal Selectivity of Myelination by Single Oligodendrocytes Established During Development in Mouse Cerebellar White Matter
Source: Glia. 2024 Dec 17;73(4):873–86. doi: 10.1002/glia.24660 (PMC11845844; doi:10.1002/glia.24660)
Supplement: Supplementary file 1 — Figure S1. [file GLIA-73-873-s001.docx]

**
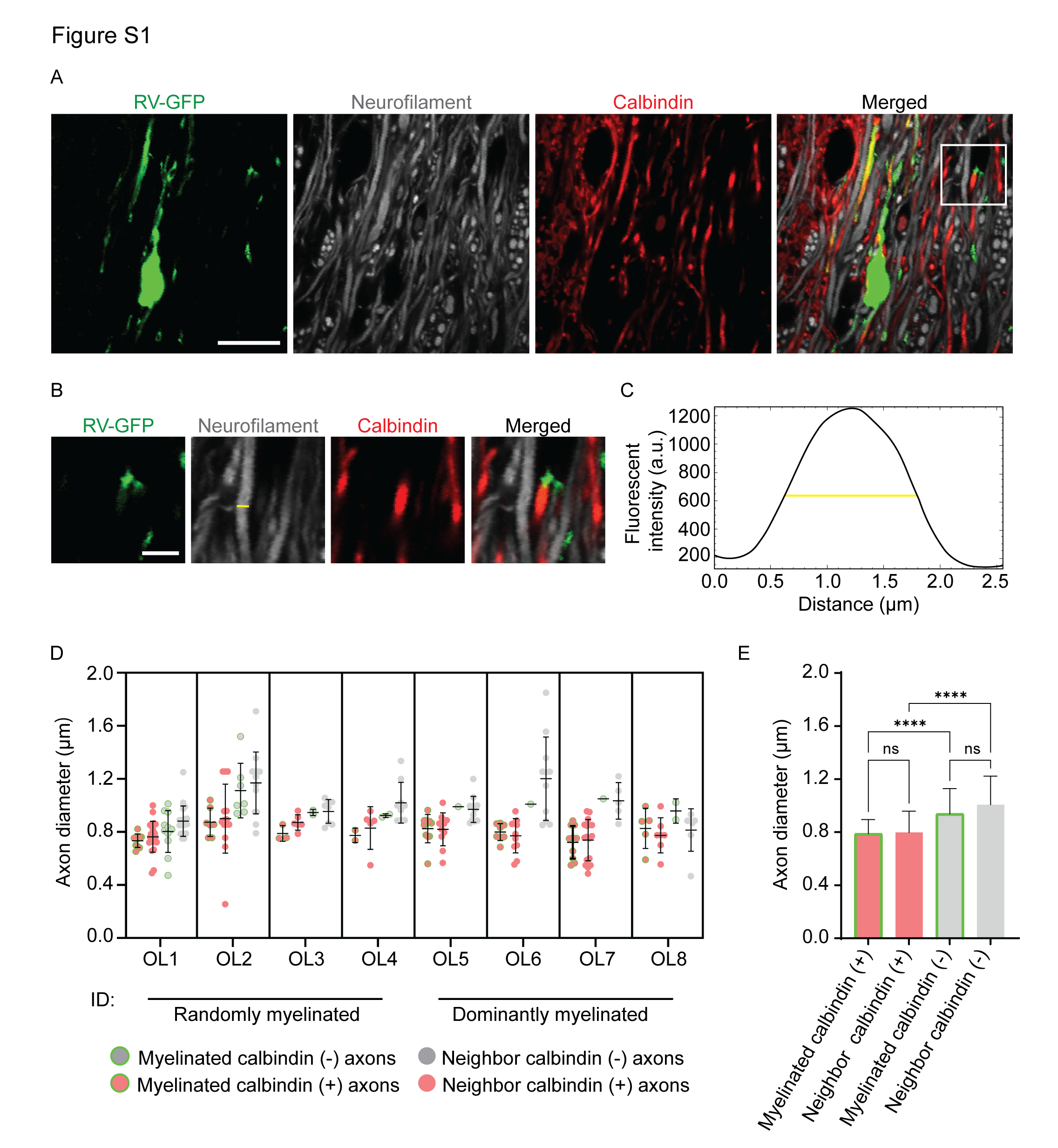
**

**Figure S1. Diameters of axons myelinated and not myelinated by labeled oligodendrocytes are similar.** (A) Representative fluorescence images of an oligodendrocyte labeled with rabies virus encoding GFP (RV-GFP) and axons immunostained for calbindin (red) and neurofilament (white). Axonal diameter is measured in the magnified images. Scale bar: 15 µm. (B) The magnified images (A, white rectangle) where the diameter analysis is performed using full width at half maximum of axonal fluorescence intensity (yellow line) (1-3). Scale bar: 5 µm. (C) A fluorescence intensity histogram of the axon indicated in the magnified image (B, yellow line). (D) The diameter of axons myelinated by single oligodendrocytes, either without any preference to classified axons (OL1-OL4, Randomly myelinated) or with preference to specific axons (OL5-OL8, Dominantly myelinated). Axons are classified as calbindin-positive / neurofilament-positive (calbindin (+)) or calbindin-negative / neurofilament-positive (calbindin (-)) axons. These axons are either myelinated by each of RV-GFP-labeled oligodendrocyte (Myelinated) or neighboring calbindin (+) and (-) axons not myelinated by the labeled oligodendrocyte (Neighbor). Each dot represents one axon, and bars show the mean ± SD. (E) The diameter of calbindin (+) and (-) axons myelinated and not myelinated by GFP-labeled oligodendrocytes. The diameter of calbindin (+) axons was tended to be smaller compared to calbindin (-) axons, regardless of whether they were Myelinated or Neighbor. ****: P <0.0001, ns: P > 0.05, one-way ANOVA with Dunn's multiple comparisons test, N = 56, 94, 27, and 85 for Myelinated calbindin (+), Neighbor calbindin (+), Myelinated calbindin (-), and Neighbor calbindin (-) axons, respectively.
